# Suppression of Fibroblast Growth Factor 23 in UMR106 Osteoblast‐Like Cells and MC3T3‐E1 Cells by Adipokine Chemerin

**DOI:** 10.1002/cbf.70051

**Published:** 2025-01-30

**Authors:** Julia Vogt, Kim Daferner, Michael Föller

**Affiliations:** ^1^ Department of Physiology University of Hohenheim Stuttgart Germany

**Keywords:** adipokine, bone, inflammation, phosphate, vitamin D

## Abstract

Endocrine fibroblast growth factor 23 (FGF23) derived from bone governs phosphate and vitamin D metabolism. Paracrine FGF23 has additional functions in different organs. Moreover, plasma FGF23 is correlated with outcomes in chronic kidney disease. FGF23 regulation is complex depending on a plethora of different factors and conditions including AMP‐dependent kinase (AMPK), inflammation, and adipokines leptin and adiponectin. Chemerin is an adipokine implicated in proinflammatory processes in adipose tissue and other organs and an activator of AMPK. Here, we investigated whether chemerin is a regulator of FGF23. UMR106 osteoblast‐like cells and MC3T3‐E1 osteoblasts were studied. Gene expression was assessed by qRT‐PCR, FGF23 protein by ELISA, and AMPK activity by western blotting. Both cell lines expressed *Cmklr1* encoding chemerin chemokine‐like receptor 1. Chemerin slightly but significantly reduced *Fgf23* expression. Chemerin reduced FGF23 protein abundance in the cell culture supernatant, and RNAi‐mediated *Cmklr1* silencing upregulated *Fgf23* expression in UMR106 cells. In the presence of AMPK inhibitor compound C, chemerin failed to suppress *Fgf23* in UMR106 cells. In conclusion, chemerin‐dependent *Cmklr1* signaling downregulates FGF23 in bone cell lines. This effect requires, at least partly, AMPK.

## Summary

Fibroblast growth factor 23 (FGF23) is an important endocrine regulator of phosphate and vitamin D metabolism and a potential disease biomarker. Additional paracrine effects control different cellular functions. The regulation of FGF23 production in bone cells is ill‐defined. Our paper uncovers chemerin, a proinflammatory adipokine, as a powerful suppressor of FGF23 production, both at transcriptional and protein level. Moreover, we demonstrate that the effect is, at least in part, dependent on AMP‐dependent kinase (AMPK). The paper adds to the emerging knowledge of FGF23 regulation required to understand the role of this endocrine and paracrine factor. Moreover, it points to the potentially important bone‐adipose tissue axis for the control of FGF23 production.

## 1 Introduction

Vitamin D and phosphate metabolism is classically under the control of parathyroid hormone (PTH) which is produced by parathyroid glands mainly following hypocalcemia, i.e. low Ca^2+^ serum concentration [1, 2]. Fibroblast growth factor 23 (FGF23) is a hormonal regulator of renal mineral metabolism that is synthesized by osteoblasts/osteocytes [3, 4]. Its main renal effects are an increase in phosphate excretion (by downregulating surface expression of renal phosphate transporter NapiIIa encoded by SLC34A1) and suppression of 1,25(OH)_2_D_3_ formation (by downregulation of 1α‐hydroxylase) [5, 6]. 1,25(OH)_2_D_3_ is active vitamin D [7].

Mechanistically, endocrine effects of FGF23 are mediated by a membrane receptor which is made up by a complex of the two transmembrane proteins FGFR1c and αKlotho [8]. αKlotho has become popular as an aging suppressor since lack of αKlotho leads to strongly accelerated aging while its overexpression results in a 30% increase in life expectancy of mice [9, 10, 11, 12, 13]. Premature aging of αKlotho deficiency is mainly due to severe changes in vitamin D and phosphate homeostasis as is FGF23 deficiency [14].

FGF23 may also have paracrine effects in other organs including heart or liver [15, 16]. In clinical medicine, FGF23 may serve as a disease biomarker [17]. First, it was shown that the FGF23 plasma concentration goes up early in the progression of renal insufficiency [18]. But also in heart and/or vessel disorders elevated FGF23 levels are positively correlated with worse outcome [17].

FGF23 synthesis is subject to complex regulation by several factors to a varying degree. Those factors include hormones (e.g. 1,25(OH)_2_D_3_ [19], PTH [20], EPO [21], insulin [22]), nutrients (e.g. phosphate [23], iron [24]), intracellular signaling (e.g. AMP‐dependent kinase signaling [25]), inflammatory pathways and cytokines (e.g. NFκB, IL‐1, IL‐6, TNF‐α) [26, 27, 28], and adipokines (e.g. leptin [29], adiponectin [30]).

Chemerin is an adipokine with proinflammatory properties and produced in white adipose tissue, but also other organs such as liver, colon, kidney, or endocrine glands express it [31, 32]. Similar to FGF23 it is subject to complex posttranslational modifications involving cleavage [33]. Chemerin is a circulating factor [34].

As an adipokine, it is a regulator of lipid metabolism and adipogenesis [35, 36]. It is relevant for frequent metabolic diseases including obesity, diabetes, and metabolic syndrome [37].

Furthermore, it interacts with different endocrine systems, regulating the secretion of gonadotropins by the pituitary gland, ovarian function, or Leydig cells in the testes thereby influencing testosterone secretion [31, 38]. It is implicated in various diseases of the reproductive system [31]. Also, bone formation is under the control of chemerin [39].

Chemerin mediates its effects mainly through chemokine‐like receptor 1 (CMKLR1) [40]. It is a powerful activator of AMPK [41] that has been demonstrated to inhibit FGF23 production [25].

Due to chemerin's emerging role in bone biology and since it influences pathways relevant for FGF23 production, we hypothesized that chemerin is a regulator of FGF23 similar to other adipokines. We performed experiments in MC3T3‐E1 mouse osteoblasts and UMR106 osteoblast‐like cells to verify this hypothesis.

## 2 Materials and Methods

### 2.1 Cell Culture

UMR106 rat osteoblastic cells (ATCC, Manassas, USA) were cultivated in Dulbecco's modified Eagle medium (DMEM) high glucose (Gibco, Life Technologies, Darmstadt, Germany) containing 100 μg/mL streptomycin, 100 U/mL penicillin, and 10% fetal bovine serum (FBS) (Gibco, Life Technologies). 2 × 10^5^ cells were seeded and kept at 37°C and 5% CO_2_. Pretreatment with 10 nM 1,25(OH)_2_D_3_ (Tocris Bioscience, Bristol, UK) was carried out. Twenty‐four hours later, they were treated with or without chemerin‐9 (149–157) (GenScript, Piscataway, NJ, USA) and with or without 1 µM AMPK inhibitor compound C (Tocris, Bio‐Techne, Wiesbaden, Germany) for further 24 h. For control, cells were exposed to the respective volume of the solvent water. Cells were only used from passages 10–26.

Murine MC3T3‐E1 pre‐osteoblast cells (subclone 4) were cultivated in alpha‐Minimum Essential Medium (α‐MEM) plus 2 mM l‐glutamine together with 10% FBS, 100 μg/mL streptomycin, 100 U/mL penicillin, and nucleosides (all from Gibco, Life Technologies). Twenty‐four hours after seeding 8 × 10^4^ cells/well on rat tail type I collagen‐coated 12‐well plates (Greiner Bio‐One), differentiation was induced by osteogenic medium (4 mM *β*‐glycerophosphate (AppliChem, Darmstadt, Germany) and 50 μg/mL ascorbic acid (Sigma‐Aldrich, Schnelldorf, Germany)). After 6 days of differentiation, cells were exposed to chemerin‐9 or vehicle plus 10 nM 1,25(OH)_2_D_3_ for 16 h. MC3T3‐E1 cells were used from passages 26–29.

### 2.2 Silencing

UMR106 cells (1.5 × 10^5^ cells/well) were cultured as previously described. After 24 h, they were transfected with 100 nM Cmklr1 small interfering RNA (siRNA) (L‐087871‐02‐0005, Dharmacon, Horizon Discovery, Cambridge, UK), or 100 nM nontargeting control siRNA (D‐001810‐10‐20; Dharmacon) together with 5 μL DharmaFECT 1 (Horizon Discovery) transfection reagent in antibiotic‐free complete medium containing 10 nM 1,25(OH)_2_D_3_. Twenty‐four hours after transfection, UMR106 cells were treated with 3 µM chemerin‐9, 250 µM 5‐aminoimidazole‐4‐carboxamide ribonucleoside AICAR (Selleckchem, München, Germany) or vehicle only for another 24 h. Finally, cells were harvested and analyzed. Silencing efficiency was determined by quantitative real‐time PCR (qRT‐PCR).

### 2.3 Quantitative Real‐Time PCR (qRT‐PCR)

RNA was isolated from UMR106 and MC3T3‐E1 cells with TriFast (Peqlab, Erlangen, Germany). For mouse *Cmklr1* transcripts, RNA from MC3T3‐E1 cells was collected with the NucleoSpin RNA Mini kit and digested with DNAse (Macherey‐Nagel, Düren, Germany). CDNA synthesis with RNA (1.2 µg; 60 ng/µL) using random primers and the GoScript Reverse Transcription System (Promega, Mannheim, Germany; 25°C for 5 min, 42°C for 1 h, and 70°C for 15 min) was the next step. qRT‐PCR was carried out with the GoTaq qPCR Master Mix (Promega) as follows: 95°C for 2 min, 39 cycles of 95°C for 10 s, annealing for 30 s, and 72°C for 25 s. The master mix of 20 µL contained 2 µL cDNA, 0.125 µM (rat *Cmklr1*), 0.25 µM (rat *Fgf23* and mouse *Tbp* and *Cmklr1*) or 0.5 µM (rat *Tbp* and mouse *Fgf23*) of a specific primer pair for the target gene, and water (for primer sequences see Table 1). TATA box‐binding protein (*Tbp*) served as a housekeeping gene, and the 2^−∆CT^ method was applied.

**Table 1:** Summary of the primer sequences used for qRT‐PCR.

Species	Gene	Forward primer (5′ → 3′)	Reverse primer (5′ → 3′)	Reference sequence	Annealing temp.
*rat*	*Tbp*	ACTCCTGCCACACCAGCC	GGTCAAGTTTACAGCCAAGATTCA	NM_001004198.1	57°C
*rat*	*Fgf23*	TAGAGCCTATTCAGACACTTC	CATCAGGGCACTGTAGATAG	NM_130754.2	57°C
*rat*	*Cmklr1*	GTGACTGATCAGCCGAGGA	CCGAGCCGTCAGAATACTCC	NM_022218.2	61°C
*mouse*	*Tbp*	CCAGACCCCACAACTCTTCC	CAGTTGTCCGTGGCTCTCTT	NM_013684.3	60°C
*mouse*	*Fgf23*	TCGAAGGTTCCTTTGTATGGA	AGTGATGCTTCTGCGACAAGT	NM_022657.5	57°C
*mouse*	*Cmklr1*	TGTGCTTCCTCGGTCTCCTA	AGCCAGGTTGACAAACCACA	NM_001359060.1	61°C

For qualitative *Cmklr1* expression analysis in UMR106 and MC3T3‐E1 cells, qRT‐PCR products (12 µL) were subjected to ethidium bromide agarose gel (1.5%) electrophoresis.

### 2.4 Cell Viability Assay

To determine viability, UMR106 cells were seeded into NUNC MicroWell 96‐Well Microplates (ThermoFisher Scientific, Roskilde, Denmark), cultured for 24 h and then were treated with 1 µM compound C or vehicle for further 24 h. Cells were then incubated for 1 h with 0.5 mg/mL MTT (3‐[4,5‐dimethylthiazol‐2‐yl]‐2,5‐diphenyltetrazolium bromide) solution (Sigma‐Aldrich, Schnelldorf, Germany). MTT solution was removed and DMSO added. Absorbance was measured at 550 nm (reference wavelength 690 nm) on a microplate reader (FLUOstar Omega; BMG Labtech, Ortenberg, Germany). Viability of compound C‐treated cells was expressed as the percentage of cell viability of vehicle‐treated cells.

### 2.5 ELISA

Cell culture supernatants of MC3T3‐E1 and UMR106 cells exposed to 3 µM chemerin‐9 for 16 h (MC3T3‐E1) or 24 h (UMR106) or vehicle only, all in the presence of 10 nM 1,25(OH)_2_D_3_, were concentrated utilizing Vivaspin 2 ultrafiltration columns (Sartorius, Göttingen, Germany). It was necessary to pretreat the cells with calcitriol for 48 h in order to reach a detectable FGF23 concentration. Mouse/rat FGF‐23 (C‐term) ELISA (Quidel, San Diego, USA) was performed.

### 2.6 Western Blotting

UMR106 cells were silenced as previously described or cells were pretreated with 10 nM 1,25(OH)_2_D_3_ for 24 h and incubated with or without 3 µM chemerin‐9 for additional 24 h. After lysis with RIPA buffer (Cell Signaling Technology, Danvers, USA) with EDTA and protease and phosphatase inhibitor cocktail (Halt, ThermoFisher Scientific), 10% SDS–PAGE was carried out (30 µg protein). Antibodies used: anti‐phospho‐AMPKα (Thr172) (40H9), anti‐AMPKα (D5A2), anti‐β‐actin (8H10D10), goat anti‐rabbit IgG HRP‐linked (Cell Signaling Technology), and goat anti‐mouse IgG HRP‐linked antibody (ab205719) (abcam, Cambridge, UK). Finally, proteins were visualized using Clarity Western (Bio‐Rad Laboratories) ECL substrate. Bands were detected by ChemiDoc MP Imaging System (Bio‐Rad Laboratories). Data are shown as ratio of (phosphorylated protein/β‐actin) over (total protein/β‐actin), normalized to control.

### 2.7 Statistics

Arithmetic means ± standard error of the mean (SEM) are given with *n* indicating the number of independent experiments. For normality testing, Shapiro–Wilk test was applied. To compare two groups, two‐tailed paired Student's *t*‐test or Wilcoxon matched‐pairs signed rank test were used, otherwise repeated measures ANOVA followed by Dunnett's or Šidák's multiple comparison test. Results with *p* < 0.05 were considered statistically significant (GraphPad Prism 10 (version 10.3.1; GraphPad Software Inc., San Diego, USA)).

## 3 Results

**Figure 1:**
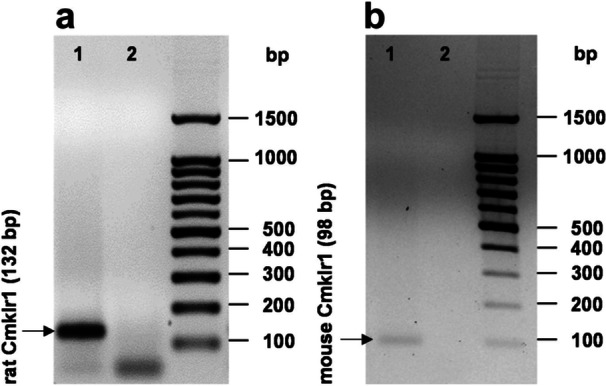
Expression of chemerin receptor *Cmklr1* in UMR106 and MC3T3‐E1 cells. Original gel photographs showing expression of *Cmklr1* (lane 1) in untreated UMR106 (a) and MC3T3‐E1 cells (b). Band size is indicated; lane 2: minus reverse transcriptase controls possibly showing primer dimers. bp, base pairs.

We performed experiments in two osteoblast cell lines, UMR106 osteoblast‐like cells and MC3T3‐E1 osteoblasts, to investigate the relevance of chemerin for FGF23. As a first step, we analyzed the expression of *Cmklr1* encoding chemerin receptor 1, the main cellular chemerin receptor [40], in the two cell lines by RT‐PCR. According to Figure 1, both UMR106 cells (Figure 1a) and MC3T3‐E1 osteoblasts (Figure 1b) expressed chemerin receptor 1.

**Figure 2:**
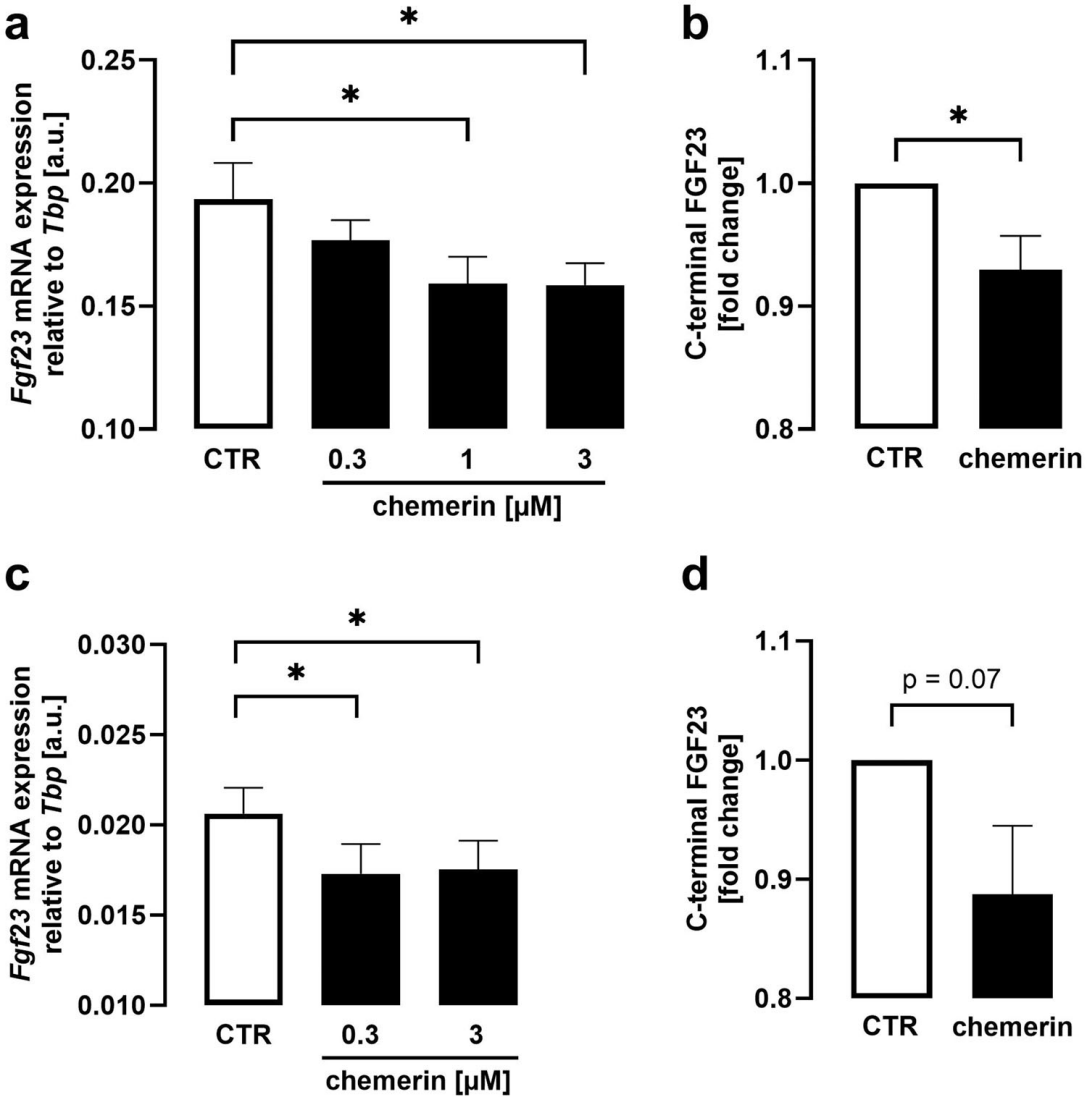
Chemerin reduced *Fgf23* gene expression and protein abundance in UMR106 and MC3T3‐E1 cells. Arithmetic means ± SEM of *Fgf23* mRNA expression relative to *Tbp* in UMR106 (a, *n* = 6) or MC3T3‐E1 cells (c, *n* = 9) incubated with vehicle control (CTR, white bars) or chemerin‐9 (black bars) at the indicated concentrations for 24 h (a) or 16 h (c). Arithmetic means ± SEM of C‐terminal FGF23 protein abundance in the supernatant of UMR106 (b) and MC3T3‐E1 (d) cells treated without (CTR) or with 3 µM chemerin‐9 for 24 h (b, *n* = 11) or 16 h (d, *n* = 13). *p < 0.05. (a, c: repeated measures one‐way ANOVA followed by Dunnett's multiple comparison test; b, d: paired *t*‐test). a.u., arbitrary units.

This result prompted us to study whether chemerin modifies *Fgf23* gene expression using qRT‐PCR. Experiments in UMR106 cells revealed that chemerin moderately but statistically significantly downregulated *Fgf23* gene expression dose‐dependently (Figure 2a). In order to find out whether this effect on gene transcription translates into reduced FGF23 protein abundance, we employed ELISA detecting C‐terminal FGF23 secreted into the supernatant. According to Figure 2b, exposure of UMR106 cells to chemerin (3 µM) led to a significantly lower FGF23 (C‐terminal) protein concentration as compared to control cells. The effect was comparable to the chemerin effect on *Fgf23* gene transcription. Next, we exposed MC3T3‐E1 cells to chemerin or vehicle and determined *Fgf23* mRNA abundance. Also in this cell line, chemerin reduced *Fgf23* gene expression (Figure 2c) and protein concentration in the cell supernatant (Figure 2d).

**Figure 3:**
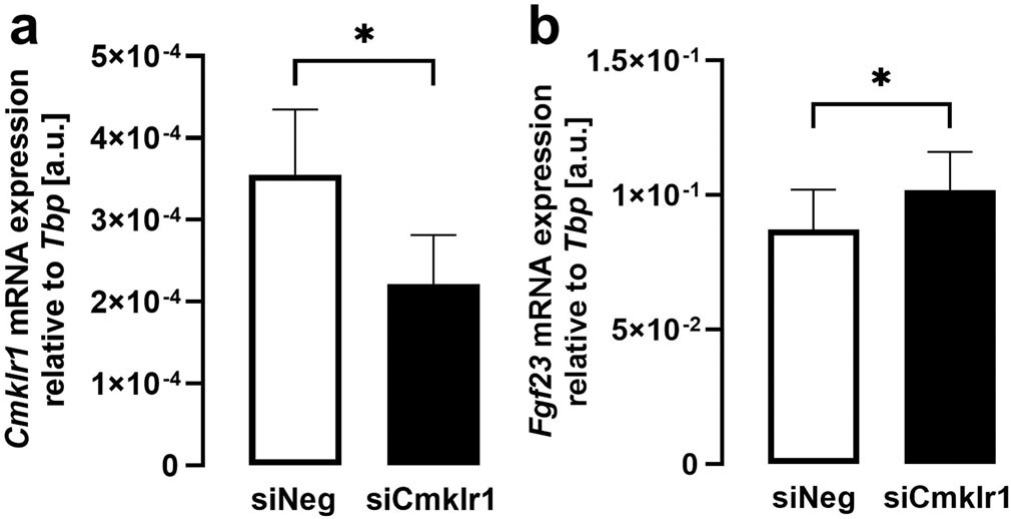
RNAi‐mediated silencing of *Cmklr1* ramped up *Fgf23* gene expression in UMR106 cells. Arithmetic means ± SEM of *Cmklr1* (a) and *Fgf23* (b) gene expression relative to *Tbp* in UMR106 cells (*n* = 11) transfected with 100 nM nontargeting siRNA (siNeg, white bars) or siRNA specifically targeting *Cmklr1* (siCmklr1, black bars) for 48 h. *p < 0.05. (a: Wilcoxon matched‐pairs signed rank test; b: paired *t*‐test). a.u., arbitrary units.

Since our experiments thus far suggest that chemerin negatively affects FGF23 in the two cell lines, we performed further experiments to elucidate the underlying signaling. As a first step, we used RNAi to verify whether chemerin receptor 1 (encoded by *Cmklr1*) is needed for *Fgf23* gene expression. As illustrated in Figure 3a, exposure of UMR106 cells to siRNA targeting *Cmklr1* was followed by a profound and significant downregulation of *Cmklr1* compared to nonsense siRNA. Importantly, siRNA‐mediated downregulation of *Cmklr1* was paralleled by a subtle, but still significant surge in gene expression of *Fgf23* in UMR106 cells (Figure 3b). These results suggest that chemerin receptor 1‐dependent chemerin signaling is a negative regulator of FGF23.

**Figure 4:**
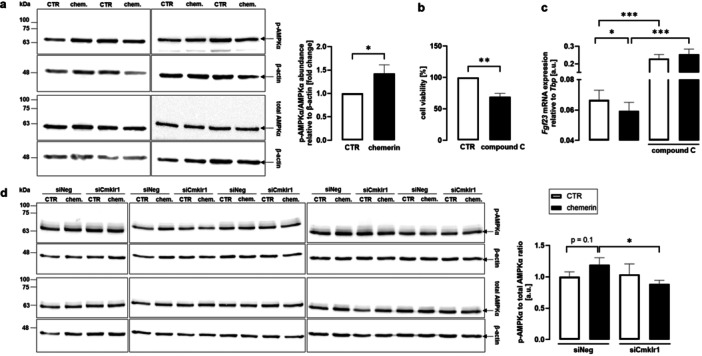
Contribution of AMPK to the chemerin‐9 effect on *Fgf23*. Representative western blots (a, left panel) and densitometric analysis (right panel; *n* = 8) of phospho‐AMPKα and total AMPKα protein, each separately over loading control β‐actin expression, in UMR106 cells treated with vehicle control (white bar) or 3 µM chemerin‐9 (black bar) for 24 h. Cell viability of UMR106 cells (b, *n* = 6) after 24‐h treatment without and with 1 µM AMPK inhibitor compound C. Arithmetic means ± SEM of *Fgf23* mRNA abundance relative to *Tbp* in UMR106 cells (c, *n* = 12) treated without (CTR, white bars) or with 3 µM chemerin‐9 (black bars) in the presence or absence of 1 µM AMPK inhibitor compound C. Representative western blots (d, left panel) and densitometric analysis (right panel, *n* = 8) of phospho‐AMPKα and total AMPKα protein, each separately over loading control β‐actin expression, in UMR106 cells transfected for 48 h with 100 nM nontargeting siRNA (siNeg) or siRNA specifically targeting Cmklr1 (siCmklr1) in the presence or absence of 3 µM chemerin‐9 for 24 h *p < 0.05, **p < 0.01, ***p < 0.001. (a, b: paired *t*‐test; c, d: repeated measures one‐way ANOVA followed by Šidák's multiple comparison test). chem., chemerin‐9; a.u., arbitrary units.

Chemerin has been demonstrated to induce AMPK [41]. Since AMPK is a potent FGF23 suppressor [25], we investigated whether AMPK is required by chemerin to suppress FGF23. Western blotting revealed that exposure of UMR106 cells to chemerin (3 µM) was paralleled by activation of AMPK as assessed by p‐AMPK protein abundance (Figure 4a). In the next series of experiments, AMPK inhibitor compound C was utilized. Treatment with compound C significantly reduced viability of UMR106 cells (Figure 4b). As expected, compound C upregulated *Fgf23* gene expression (Figure 4c). Importantly, chemerin did not significantly modify *Fgf23* in the presence of compound C, suggesting that AMPK activity was essential for the chemerin effect on *Fgf23* (Figure 4c). We next explored whether chemerin receptor 1 is required by chemerin to affect AMPK. According to Figure 4d, chemerin induced AMPK phosphorylation in UMR106 cells treated with nonsense siRNA but failed to do so in cells exposed to siRNA specifically targeting chemerin receptor (Figure 4d).

**Figure 5:**
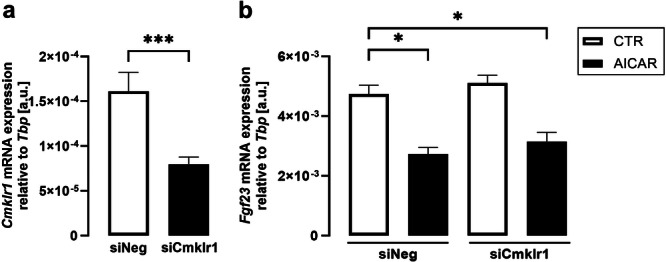
*Cmklr1* silencing did not affect the effect of AMPK activation on *Fgf23*. Arithmetic means ± SEM of *Cmklr1* (a) and *Fgf23* (b) gene expression relative to *Tbp* in UMR106 cells (*n* = 8) transfected with 100 nM nontargeting siRNA (siNeg) or siRNA specifically targeting *Cmklr1* (siCmklr1) for 48 h in the presence or absence of 250 µM AMPK activator AICAR for 24 h. *p < 0.05, ***p < 0.001. (a: paired *t*‐test; b: repeated measures one‐way ANOVA followed by Šidák's multiple comparison test). a.u., arbitrary units.

At last, we investigated whether AMPK activator AICAR abrogates the effect of silencing of chemerin receptor 1 on *Fgf23*. As displayed in Figure 5, silencing of chemerin receptor 1 was efficient (Figure 5a) and did not significantly affect the effect of AICAR on *Fgf23* (Figure 5b).

## 4 Discussion

Our study demonstrates that chemerin is a negative regulator of FGF23 in MC3T3‐E1 and UMR106 cells.

Importantly, according to our experiments in UMR106 and MC3T3‐E1 cells, chemerin reduced both, *Fgf23* mRNA levels and FGF23 protein secreted into the medium. However, despite statistical significance, the effects were numerically small. Therefore and since our study is completely based on in vitro experiments, further studies are definitely required to define the role of chemerin for FGF23 production in vivo.

Our experiments suggested that chemerin receptor 1, the main chemerin receptor, is expressed in both bone cell lines and that it participates in the regulation of FGF23 as its siRNA‐mediated knock‐down resulted in a surge of *Fgf23* transcription in UMR106 cells. This effect was observed even without exogenous chemerin. An explanation for this phenomenon could be that serum, as part of the cell culture medium, contains chemerin.

Since chemerin is generally perceived as a proinflammatory molecule [31] and inflammation is a major inducer of FGF23 [42], its negative effect on FGF23 may come as a surprise at first glance. However, chemerin is also an activator of AMPK [41], an effect which can be regarded as being anti‐inflammatory owing to AMPK's inhibitory role in proinflammatory signaling [43]. Earlier, AMPK has been demonstrated to be an important negative regulator of FGF23, a finding linking energy metabolism to FGF23 [25]. Chemerin‐dependent downregulation of FGF23 as uncovered by our study therefore appears to be plausible and broadens our knowledge of inflammation and energy metabolism being highly relevant for FGF23 regulation. Importantly, we indeed demonstrated activation of AMPK by chemerin in UMR106 cells using western blotting. Moreover, we demonstrated that this effect was dependent on chemerin receptor 1 and showed through a pharmacological approach that AMPK, at least in part, contributes to the inhibitory effect of chemerin on FGF23. Since silencing of chemerin receptor 1 did not significantly affect the effect of AICAR, AMPK can also regulate *Fgf23* independently of chemerin receptor 1. It must be, however, kept in mind that AMPK inhibitor compound C compromised cell viability.

Chronic kidney disease (CKD) is a common condition associated with early and strong surges in plasma FGF23 levels [18]. Notably, also chemerin serum levels are elevated in CKD and negatively associated with glomerular filtration rate [44]. It must be kept in mind that the chemerin effect on FGF23 appears to be small according to our cell culture study. Therefore, it appears to be likely that the effect of enhanced chemerin production on FGF23 in CKD is overridden by various other stimulating effects on FGF23 associated with CKD, e.g. phosphate overload, inflammation, hyperparathyroidism.

Adipokines have already been demonstrated to be regulators of FGF23: whereas leptin induces FGF23 production [29], adiponectin and—according to this study chemerin—are negative regulators [30, 45], pointing to a complex interplay of adipose tissue and bone in the regulation of FGF23.

Obesity is a condition characterized by higher chemerin and leptin serum levels [46]. Moreover, obese subjects typically have higher FGF23 levels, presumably due to enhanced inflammation [47]. It is tempting to speculate whether the opposing effects of the two adipokines are part of a complex adipose tissue‐derived regulatory network for FGF23.

Definitely, our study adds to the growing knowledge that chemerin is an important regulator of bone metabolism.

Taken together, we demonstrated that the adipokine chemerin is a suppressor of FGF23 in MC3T3‐E1 osteoblasts and UMR106 cells, an effect at least in part dependent on AMPK. Chemerin‐dependent FGF23 regulation may particularly play a role in states of enhanced chemerin production, i.e. obesity.

## Conflicts of Interest

Michael Föller received speaker fees from Kyowa Kirin without any relationship with this study.

## Ethics Statement

The authors have nothing to report.

## Data Availability

The data sets generated and/or analyzed during the current study are available from the corresponding author upon reasonable request.
